# Fibroblast Growth Factor 23 and Outcomes in Acute Heart Failure

**DOI:** 10.3390/biom16091362

**Published:** 2026-09-18

**Authors:** Arancha Martí-Martínez, Julio Núñez, Herminio López-Escribano, Rafael de la Espriella, Enrique Santas, Enrique Rodriguez-Borja, Òscar Miró, Pere Llorens, Aitor Alquézar-Arbé, Pablo Herrero-Puente

**Affiliations:** 1 Clinical Biochemistry Department, Valencia Clinical Hospital, 46010 Valencia, Spain; aranchamartimartinez@gmail.com (A.M.-M.); lopez_heresc@gva.es (H.L.-E.); rodriguez_enr@gva.es (E.R.-B.); 2 Cardiology Department, Valencia Clinical Hospital, Instituto de Investigación Sanitaria (INCLIVA), 46010 Valencia, Spain; rdelaespriella@incliva.es (R.d.l.E.); ensantas@gmail.com (E.S.); 3 Emergency Department, Hospital Clínic, Instituto de Investigaciones Biomédicas August Pi i Sunyer (IDIBAPS), University of Barcelona, 08036 Barcelona, Spain; omiro@clinic.cat; 4 Emergency Department, Short Stay Unit and Hospital at Home, Hospital General Dr. Balmis, Instituto de Investigación Sanitaria y Biomédica de Alicante (ISABIAL), University of Alicante, 03010 Alicante, Spain; llorens_ped@gva.es; 5 Emergency Department, Hospital de la Santa Creu i Sant Pau, 08041 Barcelona, Spain; aalquezar@santpau.cat; 6 Emergency Department, Asturias Central University Hospital, 33011 Oviedo, Spain; pabloherrero71@gmail.com; 7 Instituto de Investigación Sanitaria del Principado de Asturias (ISPA), 33011 Oviedo, Spain

**Keywords:** worsening heart failure, fibroblast growth factor 23, fibrosis, inflammation

## Abstract

Worsening heart failure (WHF) is a complex clinical syndrome associated with a high risk of mortality and further decompensations, characterized by fluid overload and upregulated inflammatory and fibrotic processes linked to organ dysfunction. Fibroblast growth factor 23 (FGF23) is an emerging proxy of fibrosis and may interact to influence long-term outcomes in WHF patients. This study evaluated the association between FGF23 levels and long-term outcomes in patients with WHF evaluated in the emergency department (ED). A total of 635 patients from the EAHFE registry, enrolled during 2007, 2009, and 2011, were prospectively included. FGF23 was measured in the first blood sample obtained at ED presentation. During a median follow-up of 380 days (interquartile range: 126 to 456), all-cause mortality occurred in 216 patients (34.0%), while 351 patients (55.3%) experienced the composite endpoint of death/WHF ED reconsultation and 295 (46.5%) experienced death/HF hospitalization. Event rates increased progressively across FGF23 levels. Patients with FGF23 above the median (48.4 pg/mL) displayed higher rates (per 100 patient-years) of all-cause mortality (49 vs. 31, *p* = 0.003), death/WHF ED reconsultation (93 vs. 65, *p* = 0.003), and death/HF hospitalization (77 vs. 56, *p* = 0.043). In continuous analyses, each doubling of FGF23 remained significantly associated with an increased risk of all three outcomes after multivariable adjustment. These associations were consistent across clinically relevant subgroups, including those defined by age, sex, and kidney function, supporting the potential prognostic value of FGF23 within this cohort, although external validation is warranted.

## 1. Introduction

Worsening heart failure (WHF) is a complex and heterogeneous clinical syndrome characterized by high mortality rates and frequent decompensation episodes, imposing a substantial clinical and socioeconomic burden worldwide [1,2]. Identifying patients at higher risk remains challenging. A defining feature of WHF is the coexistence of volume expansion and inflammatory activation [3], processes that are closely intertwined and contribute to progressive organ dysfunction [4]. These mechanisms are further linked to myocardial remodeling and organ fibrosis, which play a central role in disease progression [4]. Increasing evidence supports the concept that biomarkers integrating these pathways may provide incremental prognostic information beyond traditional measures [5]. In this context, circulating biomarkers reflecting key pathophysiological pathways, such as volume expansion, inflammation and fibrosis, have emerged as potentially valuable tools for risk stratification, often preceding overt clinical deterioration [5,6].

Fibroblast growth factor 23 (FGF23), a member of the fibroblast growth factor family, is a bone-derived hormone that regulates phosphate homeostasis by promoting phosphate excretion, suppressing calcitriol synthesis, and modulating parathyroid hormone levels [7]. Beyond its canonical role in mineral metabolism, FGF23 has emerged as a pleiotropic molecule with significant cardiovascular (CV) implications [7]. Elevated FGF23 levels rise early in chronic kidney disease (CKD) and have been consistently associated with increased CV morbidity and mortality, as well as adverse cardiac remodeling [8]. Moreover, accumulating experimental and clinical data suggest that FGF23 may actively contribute to myocardial hypertrophy and fibrosis, rather than merely reflecting disease severity [7]. In patients with heart failure (HF), circulating FGF23 concentrations are markedly elevated, especially during episodes of acute decompensation and have been linked to greater disease severity, volume expansion and clinical outcomes [8]. These findings position FGF23 as a potential integrative biomarker bridging mineral metabolism, inflammation, and adverse disease course. Against this background, the present study aimed to evaluate the prognostic significance of FGF23 in a real-world cohort of patients with WHF evaluated in the emergency department (ED). Specifically, we examined the association between FGF23 levels and long-term adverse clinical outcomes, including all-cause mortality and composite endpoints of death/WHF ED reconsultation, and death/HF hospitalization.

## 2. Materials and Methods

### 2.1. Study Sample

Of 764 consecutive patients with acute heart failure (AHF) enrolled in this EAHFE biobank substudy, 635 patients (83.1%) had adequate serum aliquot availability for simultaneous determination of circulating intact FGF23. The remaining 129 patients (16.9%) lacked biomarker measurements due to pre-analytical technical constraints (insufficient aliquot volume or hemolyzed specimens). As detailed in Table A1, excluded patients were clinically and demographically comparable to included patients, with similar event rates across all endpoints (all *p* > 0.25). This cohort has previously been used to investigate other circulating biomarkers in AHF [9].

The EAHFE registry is an ongoing Spanish multicenter observational registry enrolling consecutive patients evaluated for AHF in emergency departments from university, referral, and community hospitals [10]. To date, the EAHFE registry has completed eight phases of patient inclusion. For the present study, cases were randomly selected. AHF was diagnosed on the basis of compatible symptoms (e.g., dyspnea, orthopnea, paroxysmal nocturnal dyspnea) and signs (e.g., third heart sound, pulmonary crackles, jugular venous distension > 4 cm, resting sinus tachycardia, peripheral edema, hepatomegaly, or hepatojugular reflux) together with objective evidence of congestion; more than 95% of participants had additional diagnostic support from natriuretic peptide testing and/or echocardiography. Patients presenting with ST-elevation myocardial infarction as the primary diagnosis accompanied by AHF were excluded. Enrollment procedures and diagnostic criteria were standardized across participating centers.

In this study, patients were categorized as having worsening chronic HF (acutely decompensated HF in patients with a confirmed prior history of HF; *n* = 401, 63.1%) or new-onset (de novo) AHF (first-time presentation without prior HF history; n = 234, 36.9%). The EAHFE registry adheres to the ethical principles outlined in the Declaration of Helsinki for medical research involving human subjects. Patient recruitment periods and sample collection were approved by the Institutional Review Board of Principado de Asturias (protocol codes 160/15, 205/17, and 2022.015.) All patients provided written informed consent for participation in the registry. The study was also approved by the Institutional Review Board of Hospital Clínico Universitario de Valencia (protocol code 2024/371), approved on 3 March 2025.

### 2.2. Biomarker Measurements

Blood for biomarker assessment was obtained at the time of the initial ED evaluation. Plasma was prepared from lithium-heparin specimens and stored at −20 °C until analysis. Before FGF23 determination, samples underwent one freeze–thaw cycle. The FGF23 biomarker was subsequently analyzed at the Clinical Biochemistry and Molecular Pathology Laboratory of the University Clinical Hospital of Valencia. Physicians in charge of patient management were unaware of FGF23 levels.

The commercially available assay used was a chemiluminescence immunoassay LIAISON^®^ FGF23, for the in vitro quantitative determination of intact FGF23 polypeptide, in the LIAISON^®^-XL Analyzer (DiaSorin S.p.A., Saluggia, Italy). The assay has an analytical measurement range of 5–5000 pg/mL, a limit of detection of 5 pg/mL, and a limit of quantification of 6.5 pg/mL. Calibration was performed according to the manufacturer’s instructions and samples with concentrations exceeding the analytical measurement range were diluted 1:10 and reanalyzed according to the assay procedure. All samples were analyzed in a single batch. The intact FGF23 assay, which exclusively recognizes the complete and active FGF23 molecule, better reflects its biological activity compared to the carboxy-terminal form, which detects both the active form and C-terminal fragments and reflects the bone transcription of FGF23 [11]. The mean values of the quality control for FGF23 were as follows: Level 1 = 149.8 pg/mL and Level 2 = 570.5 pg/mL. The total variation coefficient for the assay, as indicated by the manufacturer, was 2.8% for both control levels. FGF23 was categorized based on the median of the overall sample (48.4 pg/mL) and into quartiles (Q1 ≤ 27.3 pg/mL; Q2 = 27.4–48.4, Q3 = 48.5–88.2; Q4 > 88.2). Two patients exhibited values above 1000 pg/mL. In both cases patients showed estimated glomerular filtration rate (eGFR) lower than 20 mL/min/1.73 m^2^.

### 2.3. Endpoints

Three time-to-event outcomes were prespecified for the present analysis: all-cause mortality, the composite of all-cause death or WHF ED reconsultation, and the composite of all-cause death or HF hospitalization. WHF events comprised an ED reconsultation or an unplanned HF hospitalization for clinical deterioration requiring intravenous diuretics or inotropic therapy. Outcomes were ascertained from electronic medical records and outpatient follow-up. Investigators assessing endpoints were blinded to FGF23 concentrations.

### 2.4. Statistical Analysis

Continuous data were summarized using mean ± SD for approximately normally distributed variables and median (25th–75th percentile) otherwise; categorical data are reported as counts and percentages. Because no clinically established FGF23 cutoff exists in AHF, the biomarker was examined primarily on a continuous scale and, for descriptive purposes, according to its median (48.4 pg/mL) and quartiles. Between-group comparisons used ANOVA or the Kruskal–Wallis test for continuous variables, as appropriate, and the χ^2^ test for categorical variables. Incidence rates (IR) for clinical outcomes were calculated as the number of first events divided by the total accrued person-time at risk in years and expressed as the number of events per 100 person-years (P-Y), with 95% confidence intervals based on the Poisson distribution Associations between the exposure (FGF23) and time-to-event outcomes were estimated using multivariable Cox proportional hazards regression.

Potential confounders were identified a priori on clinical grounds and from previous evidence rather than according to their univariable associations with the outcomes. Model reduction was subsequently performed using backward elimination with *p* < 0.20 as the retention criterion. Variables reflecting clinical congestion were kept in the models irrespective of their statistical contribution to ensure adjustment for the burden of volume overload. The resulting adjustment set comprised age, sex, previous HF, ischemic heart disease, atrial fibrillation at presentation, systolic blood pressure, heart rate, Barthel index, orthopnea, paroxysmal nocturnal dyspnea, third heart sound, jugular venous distension, pleural effusion, peripheral edema, hemoglobin, NT-proBNP, and creatinine.

Collinearity by Variance Inflation Factors (VIF) was verified for all predictors (mean VIF = 1.15; maximum = 1.34), ruling out multicollinearity. In complementary analyses, multivariable fractional polynomials (MFP) were used to test for non-linear functional forms. A retention threshold of *p* < 0.20 was used during MFP variable selection, which asymptotically approximates the Akaike Information Criterion (AIC) and prevents underfitting and coefficient bias; FGF23 was forced into all models. The proportional hazards assumption was verified via Schoenfeld residual tests (global *p* > 0.05 across all endpoints). Incremental predictive performance of FGF23 beyond the base multivariable model was assessed using Harrell’s C-concordance statistic and likelihood-ratio tests. In addition, the Integrated Discrimination Improvement (IDI) and category-free (continuous) Net Reclassification Improvement (NRI) were calculated with corresponding 95% confidence intervals (95% CI) to assess discrimination slopes and reclassification performance [12]. Estimates of risk were presented as a continuous variable after logarithmic transformation with base 2 (log_2_ FGF23). In this formulation, hazard ratios (HRs) directly reflect the relative risk associated with a doubling of circulating FGF23 levels. For standardized comparison across biomarkers, effect estimates were also expressed per 1-SD increase in log_2_ (“FGF23”).

To assess potential selection bias arising from unavailable biomarker samples (n = 129, 16.9% of the 764 cohort participants, due to insufficient plasma volume or hemolysis), a secondary sensitivity analysis was conducted using Multiple Imputation by Chained Equations (MICE). Under the assumption of missing at random (MAR), log_2_-transformed FGF23 was imputed across 10 datasets using linear regression conditional on all baseline covariates as well as the survival time and event indicators [13], Cox proportional hazards models were fitted across imputed datasets and combined using Rubin’s rules. Complete-case analysis (N = 635) was retained as the primary analytical framework.

To address the competing risk of all-cause death for nonfatal outcomes (WHF ED reconsultation and HF hospitalization), both cause-specific Cox models (censoring at competing death to evaluate etiological HRs) and Fine-Gray subdistribution hazard models (accounting for competing death to evaluate associations with the cumulative incidence function) were fitted.

In the analysis stratified by subgroups, renal function was evaluated as continuous eGFR using the CKD-EPI formula and classified into three clinically established strata: preserved/mild impairment (eGFR ≥ 60 mL/min/1.73 m^2^), moderate impairment (eGFR 30–59.9 mL/min/1.73 m^2^), and severe impairment (eGFR < 30 mL/min/1.73 m^2^). Potential effect modification between FGF23 and renal function was formally tested by incorporating multiplicative cross-product terms in multivariable flexible parametric models, evaluated via 2-degree-of-freedom Wald tests. Stratum-specific adjusted HRs and 95% CI were obtained through linear combinations.

A two-sided *p*-value of <0.05 was considered the threshold for statistical significance. All analyses were conducted using STATA version 16.1 [Stata Statistical Software, Release 16 (2019); StataCorp LP, College Station, TX, USA].

## 3. Results

### 3.1. Baseline Characteristics of the Whole Sample Stratified by the FGF23 Median

The mean ± SD age of the population was 82.4 ± 10 years, and 339 (53.4%) participants were women. A prior history of hypertension was highly prevalent (84.1%), and 401 patients (63.1%) had a previous diagnosis of HF. The median (p25% to p75%) creatinine, hemoglobin and NT-proBNP were 1.2 mg/dL (0.9–1.6), 12 g/dL (10.6–13.4), and 4207 pg/mL (2280–8421), respectively. Regarding exposure, the median (p25% to p75%) of FGF23 was 48.4 pg/mL (27.3–88.2). The baseline clinical characteristics of the study cohort stratified according to median FGF23 levels are summarized in Table 1.

Patients with FGF23 concentrations above the median showed higher rates of CKD and ischemic heart disease. These individuals also showed a greater burden of fluid overload, with more frequent pleural effusion and jugular venous distension, and exhibited lower left ventricular ejection fraction (LVEF). Also, those above the median showed higher NT-proBNP and lower eGFR and hemoglobin. Overall, these patients had a worse baseline risk profile, reflected by a higher MEESSI risk score. Similar differences were also found when assessing FGF23 quartiles (Table A2).

### 3.2. Adverse Clinical Events

At a median follow-up of 380 days (25th–75th percentile, 126–456), 216 patients (34.0%) died from any cause, 233 (36.7%) experienced a non-fatal WHF ED reconsultation (median time to event: 29.0 days, IQR: 16.0–47.0), and 152 (23.9%) had a non-fatal HF hospitalization (median time to event: 27.5 days, IQR: 15.0–42.5). At median follow-ups of 362 days (44–442) and 354 days (40–435), 351 patients (55.3%) experienced the composite endpoint of death/WHF ED reconsultation, and 295 patients (46.5%) experienced death/HF hospitalization, respectively. The corresponding incidence rates per 100 person-years were 39 (95% CI, 35–45) for all-cause mortality, 78 (95% CI, 70–87) for death/WHF ED reconsultation, and 66 (95% CI, 59–74) for death/HF hospitalization.

### 3.3. Relationship Between FGF23 and Adverse Clinical Events

**Figure 1:**
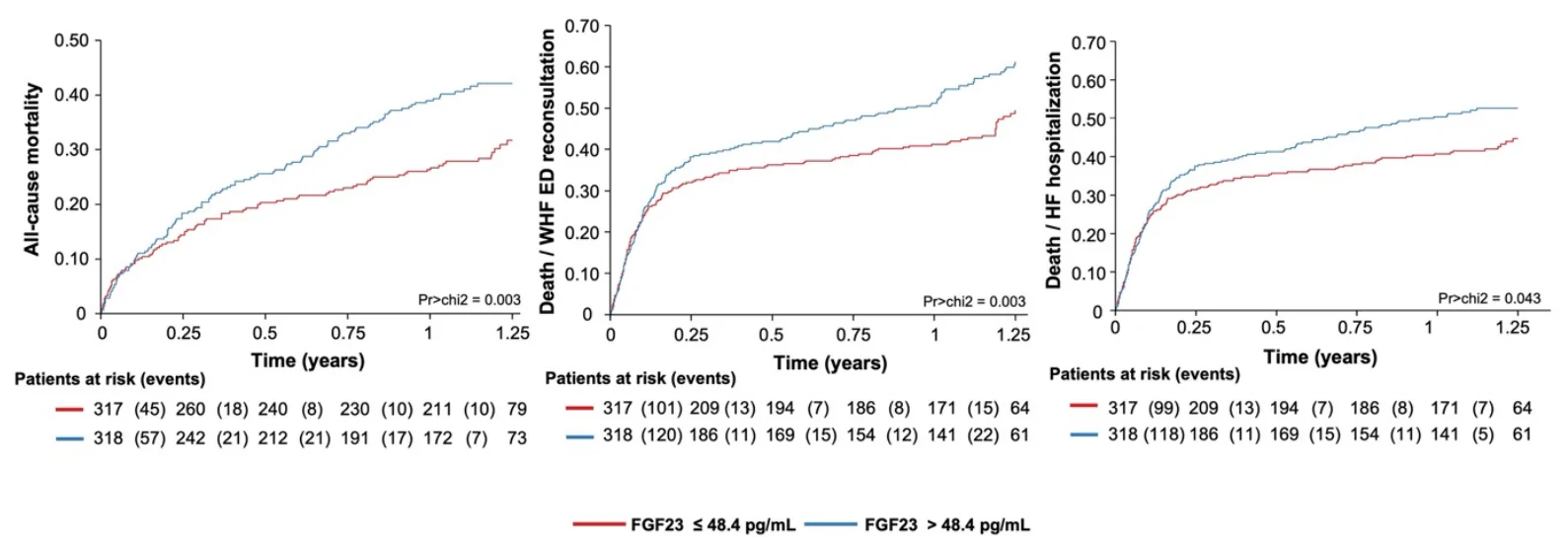
Kaplan–Meier curves for adverse clinical events across the FGF23 median. Cumulative all-cause mortality, death/WHF ED reconsultation and death/HF hospitalization over 1.25 years are shown for patients stratified by median of FGF23. Patients at risk and number of events for each time point are displayed below each panel. FGF23 = fibroblast growth factor 23; HF = heart failure; ED = emergency department; WHF = worsening heart failure.

**Figure 2:**
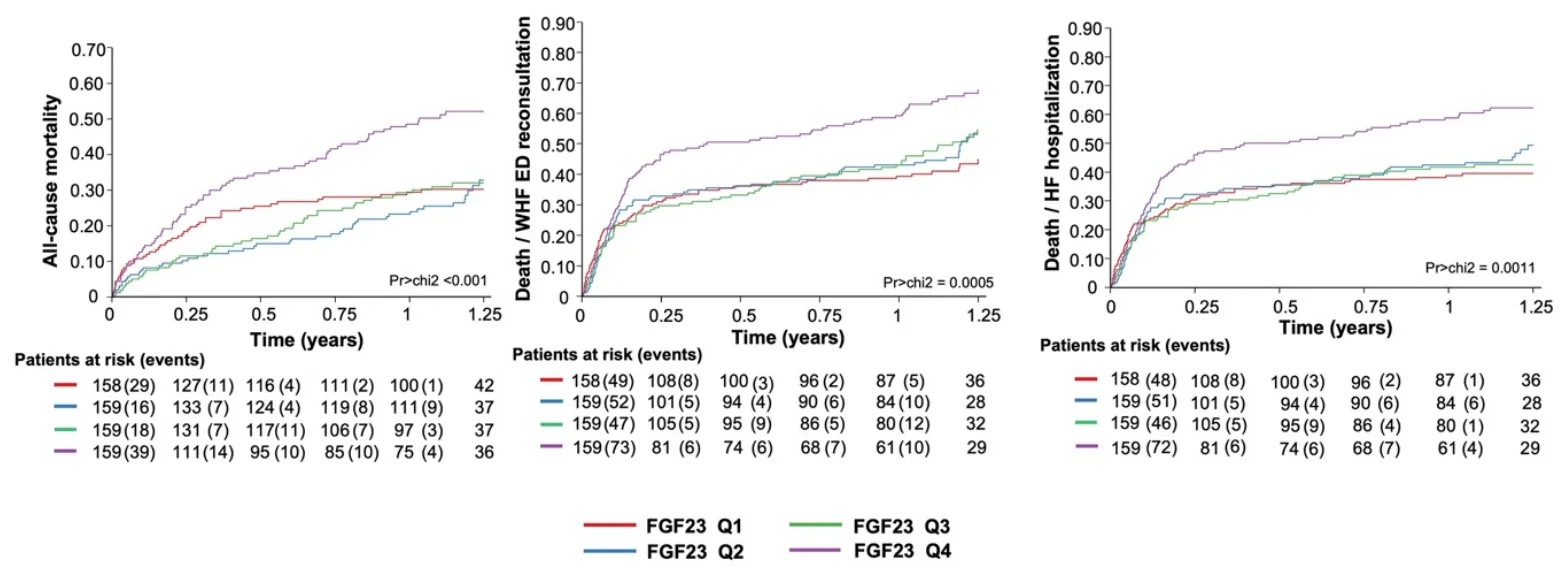
Kaplan–Meier curves for adverse clinical events across FGF23 quartiles (Q1–Q4). Cumulative all-cause mortality, death/WHF ED reconsultation and death/HF hospitalization over 1.25 years are shown for patients stratified by FGF23 quartiles. Patients at risk and number of events for each time point are displayed below each panel. FGF23 = fibroblast growth factor 23; HF = heart failure; ED = emergency department; WHF = worsening heart failure.

Patients with FGF23 concentrations above the median (>48.4 pg/mL) exhibited higher incidence rates across all three adverse outcomes (Table 1). When stratified by quartiles, individuals in the highest quartile (≥88.2 pg/mL) had nearly a two-fold increase in event rates compared with those in the lowest quartile (≤27.2 pg/mL) (Table A3). Kaplan–Meier analyses showed a consistent pattern across all three endpoints. Patients with FGF23 concentrations above the median had a significantly higher cumulative risk of all-cause mortality, death/WHF ED reconsultation, and death/HF hospitalization, with curves diverging after 2–3 months (Figure 1; all *p*-values < 0.05). Likewise, Kaplan–Meier curves stratified by FGF23 quartiles displayed a clear, graded association, with progressively worse outcomes across increasing quartiles (Figure 2; all *p*-values < 0.05).

**Figure 3:**
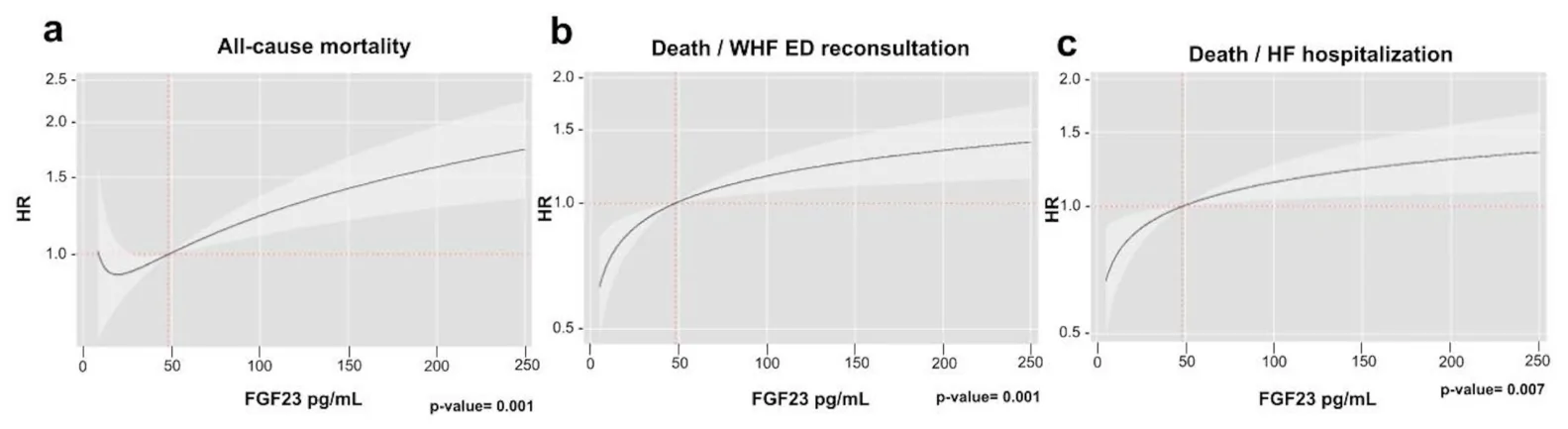
FGF23 and its association with adverse clinical outcomes expressed as adjusted hazard ratios. (**a**) All-cause mortality; (**b**) death/WHF ED reconsultation; (**c**) death/HF hospitalization. HF = heart failure; HR = hazard ratio; FGF23 = fibroblast growth factor 23; ED = emergency department; WHF = worsening heart failure. The *x*-axis is truncated at the 97th percentile of FGF23 to optimize visual resolution of the cohort; all patients (N = 635) were included in model estimation.

In the overall cohort, multivariable analyses showed a consistent and independent association between higher FGF23 concentrations and increased risk of all three endpoints (all *p*-values < 0.05). When modeled as a continuous variable using fractional polynomials, FGF23 exhibited a non-linear association with all clinical outcomes. For all-cause mortality (Figure 3a), we found a progressive and nearly linear increase in hazard above 40–50 pg/mL. At higher concentrations, the risk increased steadily, reaching HRs approaching ~1.7–1.8 at the upper range of the distribution (*p* = 0.001). Each doubling of FGF23 was independently associated with a 17% higher risk of all-cause mortality (adjusted HR: 1.17, 95% CI: 1.05–1.31; *p* = 0.004; equivalent to adjusted HR: 1.24, 95% CI: 1.07–1.43 per 1-SD increase).

For death/WHF ED reconsultation (Figure 3b) the adjusted HRs rose sharply from lower until values about 50–60 pg/mL with a more gradual increase at higher concentrations (*p* = 0.001). A similar pattern was observed for death/HF hospitalization (Figure 3c), where the association followed a comparable trajectory, with an early steep rise and subsequent attenuation of the slope, although risk continued to increase across the full range of FGF23 values (*p* = 0.007). Similarly, each doubling of FGF23 independently predicted an increased hazard of death/WHF ED reconsultation (adjusted HR: 1.14, 95% CI: 1.05–1.24; *p* = 0.002; adjusted HR: 1.20, 95% CI: 1.07–1.34 per 1-SD increase) and death/HF hospitalization (adjusted HR: 1.12, 95% CI: 1.02–1.22; *p* = 0.013; adjusted HR: 1.17, 95% CI: 1.03–1.31 per 1-SD increase). Risk estimates across median, quartiles, per doubling (log2 scale), and per 1-SD increase in log2 are presented in Table 2.

As a sensitivity analysis, we additionally fitted a clinically defined fully saturated model including all candidate covariates simultaneously, without variable elimination. The adjusted effect estimates for FGF23 remained virtually unchanged compared with those obtained using the MFP-selected model, supporting the robustness of the association irrespective of the variable-selection strategy (Table A4).

In a sensitivity analysis evaluating the full registry cohort (*N* = 764) using multiple imputations by chained equations (10 imputed datasets) to account for the 129 patients with unmeasured biomarker aliquots, the independent prognostic association of circulating FGF23 remained fully consistent with primary complete-case models. After adjusting for all covariates, each doubling of FGF23 was independently associated with an increased hazard of all-cause mortality (adjusted HR: 1.18, 95% CI: 1.06–1.31; *p* = 0.003), death/WHF ED reconsultation (adjusted HR: 1.12, 95% CI: 1.03–1.22; *p* = 0.006), and death/HF hospitalization (adjusted HR: 1.11, 95% CI: 1.02–1.21; *p* = 0.014).

When evaluating the incremental value of FGF23, our findings show that incorporating circulating FGF23 results in statistically significant improvements in overall model fit (by likelihood-ratio tests) and IDI across all three clinical endpoints, alongside significant net reclassification for all-cause mortality and ED reconsultations (Table 3).

### 3.4. Association of FGF23 with Adverse Clinical Events Stratified by Subgroups

As exploratory analyses, we assessed the association between FGF23 and age, sex and kidney function subgroups. Risk estimates among subgroups are presented in Table 4.

#### 3.4.1. Age

**Figure 4:**
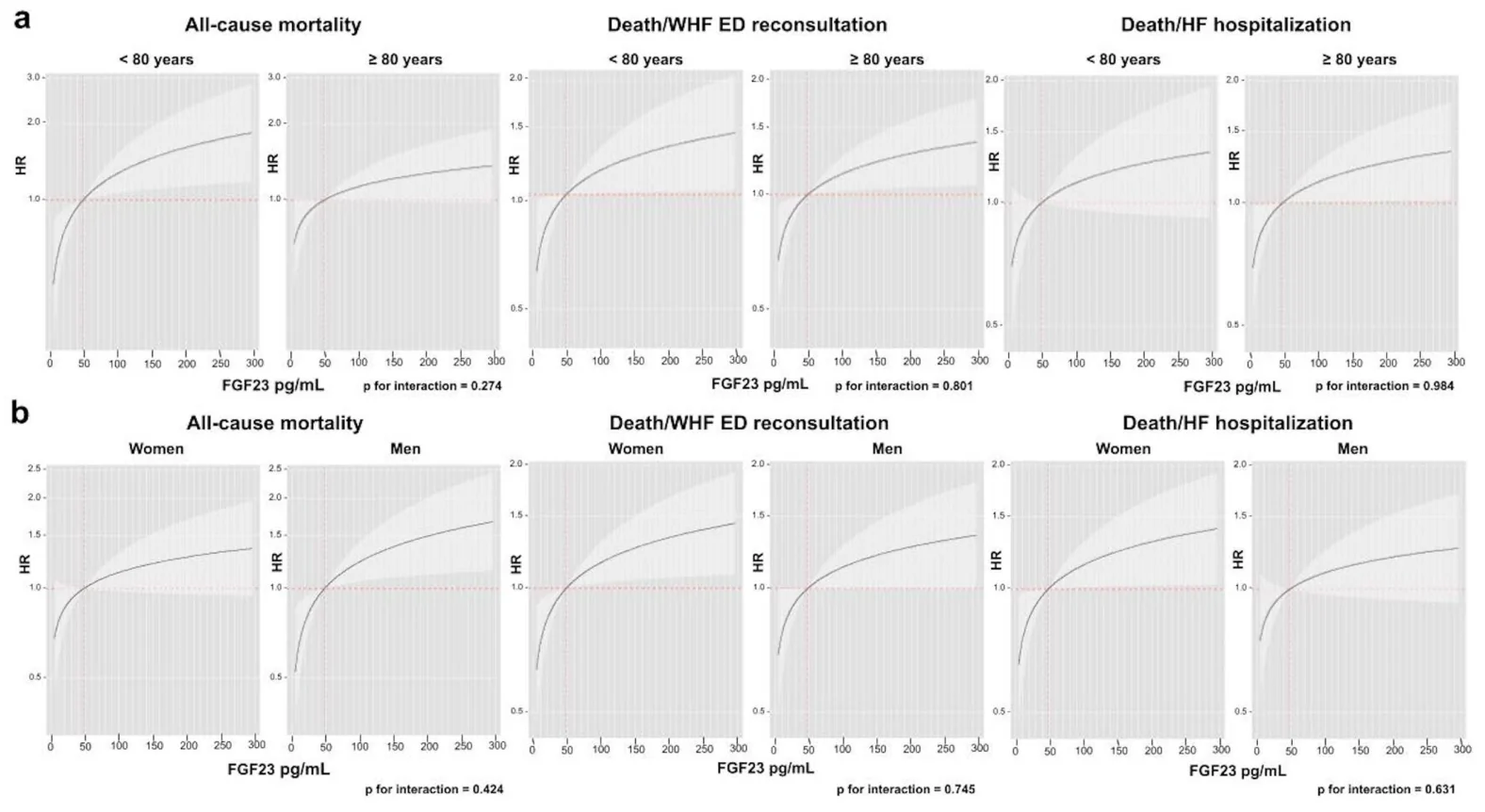
Hazard ratio depiction for all-cause mortality, death/WHF ED reconsultation and death/HF hospitalization along the continuum of FGF23 in the multivariable model stratified by age and sex. (**a**) Age (**b**) Sex. HF = heart failure; HR: hazard ratio; FGF23 = fibroblast growth factor 23; ED = emergency department; WHF = worsening heart failure. The *x*-axis is truncated at the 97th percentile of FGF23 to optimize visual resolution of the cohort; all patients (N=635) were included in model estimation.

When stratified by age, the association between FGF23 and adverse outcomes was qualitatively similar in patients aged <80 and ≥80 years across all endpoints (all-cause mortality, death/HF hospitalization and death/WHF ED reconsultation). In both age groups, higher FGF23 concentrations were associated with a progressive increase in risk, following a comparable non-linear pattern characterized by an early rise and subsequent attenuation at higher levels (Figure 4a). Formal interaction testing showed no evidence of effect modification by age for any outcome (all-cause mortality: *p* for interaction = 0.274; death/WHF ED reconsultation: *p* for interaction = 0.801; death/HF hospitalization: *p* for interaction = 0.984), indicating no significant heterogeneity across age categories.

#### 3.4.2. Sex

When stratified by sex, the association between FGF23 and adverse outcomes was broadly similar in women and men across all endpoints (Figure 4b). Formal interaction testing showed no evidence of effect modification by sex for any outcome (all-cause mortality: *p* for interaction = 0.424; death/WHF ED reconsultation: *p* for interaction = 0.745; death/HF hospitalization: *p* for interaction = 0.613), indicating no significant heterogeneity between women and men.

#### 3.4.3. Kidney Function

**Figure 5:**
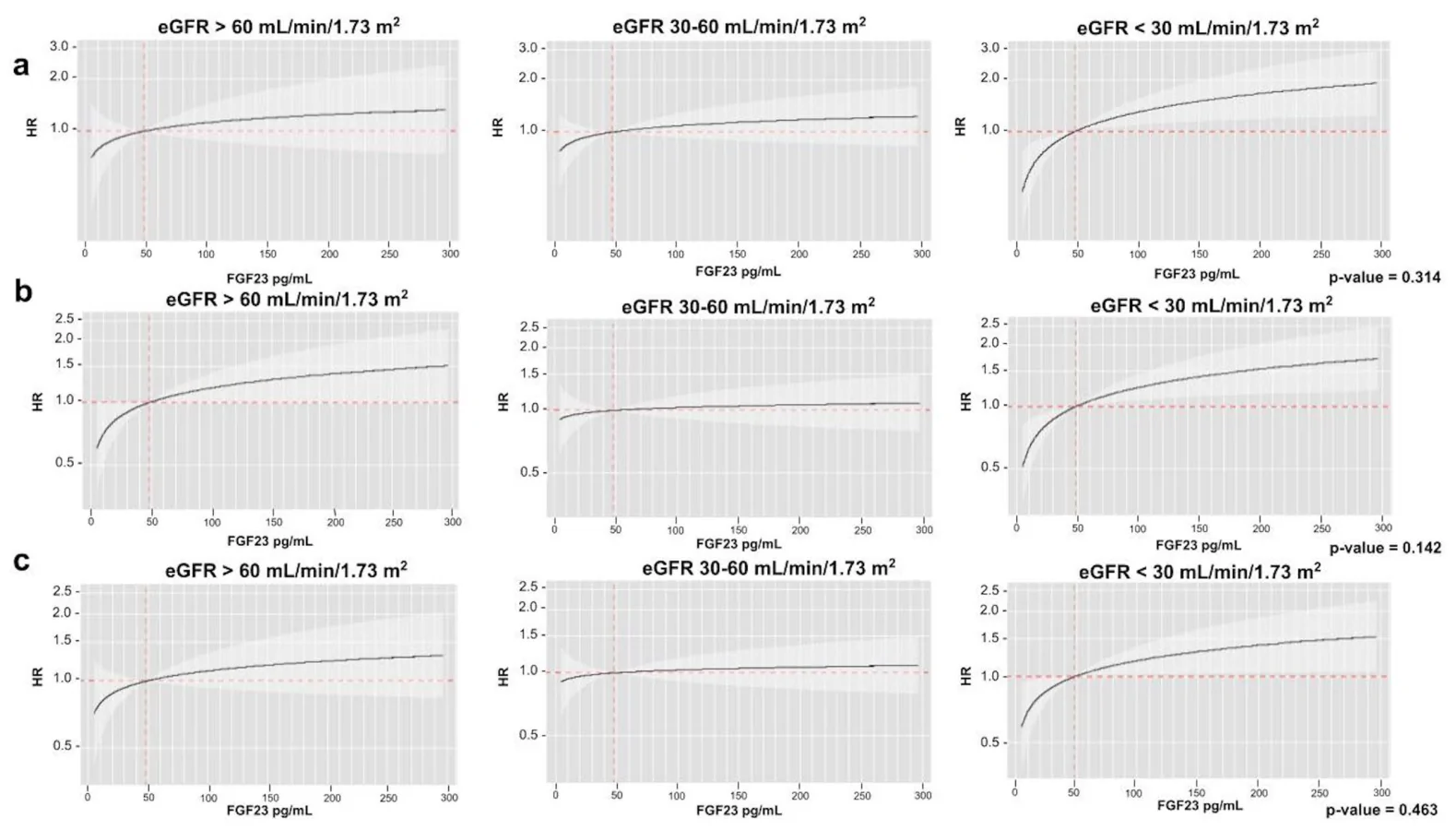
Hazard ratio depiction for all-cause mortality, death/WHF ED reconsultation and death/HF hospitalization along the continuum of FGF23 in the multivariable model stratified by eGFR. (**a**) All-cause mortality; (**b**) death/WHF ED reconsultation; (**c**) death/HF hospitalization. HF = heart failure; HR = hazard ratio; FGF23 = fibroblast growth factor 23; ED = emergency department; WHF = worsening heart failure. The *x*-axis is truncated at the 97th percentile of FGF23 to optimize visual resolution of the cohort; all patients (N = 635) were included in model estimation.

When stratified by baseline kidney function, the association between FGF23 and adverse outcomes was qualitatively consistent across eGFR categories for all endpoints (all-cause mortality, death/WHF ED reconsultation, and death/HF hospitalization; Figure 5a–c). Higher FGF23 concentrations were associated with a progressive increase in risk across all strata, with a similar non-linear pattern characterized by an early rise followed by a more gradual increase at higher levels.

Although the slope of the association appeared numerically steeper among patients with more advanced renal dysfunction (eGFR < 30 mL/min/1.73 m^2^), formal interaction testing showed no evidence of statistically significant interaction by renal function for any of the outcomes (all-cause mortality: *p* for interaction = 0.314; death/WHF ED reconsultation: *p* for interaction = 0.142; death/HF hospitalization: *p* for interaction = 0.463), indicating no significant heterogeneity across eGFR categories.

### 3.5. FGF23 and Risk of Isolated Endpoints

When evaluated as isolated endpoints, each doubling of circulating FGF23 was independently associated with all-cause mortality (adjusted HR 1.17, 95% CI: 1.05–1.31; *p* = 0.004). When nonfatal outcomes were evaluated separately accounting for the competing risk of death, the multivariable-adjusted associations were modest and did not reach statistical significance: the Fine–Gray subdistribution hazard ratio (sHR) was 1.08 (95% CI: 0.97–1.20; *p* = 0.141) for WHF ED reconsultation and 1.07 (95% CI: 0.95–1.21; *p* = 0.285) for HF hospitalization. Cause-specific Cox models yielded concordant estimates (csHR 1.09; *p* = 0.123 and csHR 1.07; *p* = 0.298, respectively).

## 4. Discussion

In this post hoc biomarker analysis of the EAHFE registry, higher circulating FGF23 levels were independently associated with an increased risk of long-term adverse outcomes in patients with WHF attended in the ED. Interestingly, higher FGF23 was associated not only with mortality but also with the composite outcome of death or WHF ED reconsultation during follow-up. These associations remained consistent after comprehensive adjustment for key confounders, including age, sex, and kidney function, and across clinically relevant subgroups, including kidney dysfunction. These findings, consistent with previous evidence [14,15], further support an association between FGF23 and adverse clinical outcomes in HF, especially mortality, and suggest its potential value as a prognostic biomarker in this high-risk setting.

Although the absolute increase in the C-statistic was small, this is expected when evaluating a novel biomarker against a well-performing multivariable base model. While the C-statistic is often relatively insensitive to the addition of new predictors in multivariable models [16], the observed improvements in likelihood-ratio tests and IDI across the three endpoints, together with significant net reclassification for all-cause mortality and death/WHF ED reconsultation, provide statistical evidence of incremental prognostic performance of FGF23 beyond established cardiorenal risk factors. However, these findings should not be interpreted as evidence of clinical utility, which requires independent external validation and assessment of its impact on clinical decision-making.

Importantly, when nonfatal outcomes were considered separately, the associations with FGF23 were modest and did not reach statistical significance. Fine–Gray subdistribution hazard models, which characterize the association with the cumulative incidence of nonfatal events in the presence of competing death, and cause-specific Cox models, which estimate the instantaneous event rate among individuals remaining at risk, yielded concordant results. These findings suggest that the associations observed for the composite endpoints may be driven predominantly by mortality and should not be interpreted as evidence of a significant independent association between FGF23 and isolated nonfatal events.

### 4.1. FGF23 and WHF: Prior Evidence

Our findings extend previous evidence linking FGF23 to adverse outcomes in cardiovascular and cardiorenal populations [17]. Beyond its role as a biomarker of mineral metabolism, FGF23 has been increasingly recognized as a key player in several pathophysiological mechanisms involved in HF progression [14]. Experimental studies have demonstrated that FGF23 directly induces left ventricular hypertrophy [18], and promotes sodium retention and plasma volume expansion through enhanced distal tubular sodium reabsorption [19], thereby providing a plausible mechanistic link with congestion and WHF.

In chronic HF, a substantial body of evidence has consistently shown that higher FGF23 concentrations are associated with greater disease severity and worse prognosis. Elevated levels correlate with poorer functional status, impaired renal function, and increased mortality risk [20], and have been identified as an independent predictor of death in patients with HFrEF [21]. This relationship has also been described in HFpEF [22,23], where FGF23 is associated with reduced functional capacity and adverse outcomes, supporting its role across the full spectrum of HF phenotypes.

Evidence is also available in patients with AHF and WHF. In patients with new-onset or WHF, elevated FGF23 levels have been associated with greater congestion, poorer optimization of guideline-directed medical therapy, and an increased risk of mortality and HF-related hospitalizations [8]. Similarly, in hospitalized acute HF cohorts, early measurement of intact FGF23 has been associated with disease severity and increased one-year mortality, with prognostic performance comparable to established risk prediction models [24]. In more severe forms of decompensation, including cardiogenic shock, higher FGF23 levels have also been linked to short-term mortality [25].

In this context, our study provides additional insights. First, we found that FGF23 measured in patients evaluated in the emergency department, rather than exclusively in selected hospitalized cohorts, offers prognostic information from the earliest stages of acute decompensation. This finding is particularly important, as early risk stratification in the emergency setting remains challenging and may help guide decisions regarding patient disposition and intensity of follow-up [26]. Second, our population was very elderly, a group frequently underrepresented in biomarker studies [27] despite representing a large proportion of patients presenting with acute HF in routine clinical practice. Third, our cohort included a broad spectrum of HF phenotypes, reflecting the heterogeneity encountered in routine clinical practice. Finally, the observed association with WHF and HF-related admissions is especially relevant, as it suggests that FGF23 may identify patients at risk of subsequent clinical instability beyond its previously established relationship with mortality. Altogether, our findings support the concept that FGF23 reflects not only cardiorenal dysfunction but also congestion-related pathophysiological pathways driving recurrent decompensation.

From a mechanistic perspective, the clinical associations observed in our study are also supported by a solid body of experimental evidence. FGF23 is a phosphaturic hormone central to mineral metabolism, regulating phosphate homeostasis and suppressing active vitamin D synthesis [14,28], by inhibiting renal phosphate reabsorption and downregulating 1α-hydroxylase while upregulating 24-hydroxylase [29]. Its canonical actions occur in the kidney and parathyroid glands via FGFR–klotho signaling [29]. Beyond its role in mineral metabolism, FGF23 has emerged as a direct modulator of CV damage [14]. At the myocardial level, klotho-independent activation of FGFR4 triggers the PLCγ–calcineurin–NFAT pathway [30], increasing intracellular calcium and promoting hypertrophic gene expression [31]. Concurrently, it activates pro-fibrotic (β-catenin, TGF-β/Smad) and pro-inflammatory pathways, enhancing extracellular matrix production and cytokine release, thereby contributing to myocardial fibrosis, inflammation, and adverse remodeling [7,32]. In parallel, FGF23 may be integrated within the congestion-inflammation axis that characterizes most episodes of WHF [33]. Volume expansion promotes systemic inflammatory activation [34], with cytokines such as TNF-α, IL-1β, and IL-6, which have been shown to induce FGF23 expression [7]. Thus, elevated FGF23 levels may further amplify myocardial remodeling, perpetuating cardiac dysfunction and congestion [29], suggesting an active role within this vicious cycle. Furthermore, beyond the myocardial effects, FGF23 may exert indirect cardiovascular actions through neurohormonal activation [7]. By reducing calcitriol levels, FGF23 disinhibits renin expression and activates the renin–angiotensin–aldosterone system, resulting in increased angiotensin II and aldosterone activity [7,30]. This neurohormonal cascade further amplifies key pathological processes, including hypertrophy, fibrosis, inflammation, and hypertension [30]. Collectively, these mechanisms support the concept that FGF23 is not merely a biomarker of disease severity but may also function as an active mediator of CV damage [34].

In summary, our results support the potential role of FGF23 as an early prognostic biomarker in acute and WHF, particularly in elderly patients evaluated in the ER. Future studies should determine whether incorporating FGF23 into multimarker strategies improves risk stratification and whether serial measurements may better capture the dynamic changes associated with decompensation and treatment.

### 4.2. Potential Clinical Implications

From a clinical perspective, these results suggest the potential utility of FGF23 as a biomarker for risk stratification in patients with WHF. Its association with adverse outcomes across multiple subgroups supports its potential value for prognostic assessment, although further studies and external validation are needed before its incorporation into clinical risk models. Moreover, previously proposed links between FGF23 and cardiac remodeling raise the hypothesis that FGF23 or its downstream signaling pathways could represent potential therapeutic targets. However, our observational findings do not establish a causal or mechanistic role for FGF23, and this possibility requires further investigation in experimental and interventional studies.

### 4.3. Limitations

There are several limitations worthy of mention. First, the possibility of residual confounding cannot be excluded, as some relevant variables were not measured. In addition, echocardiographic parameters other than LVEF, as well as klotho and other mineral-metabolism variables were not collected. Therefore, despite adjustment for measured markers of renal function, residual confounding related to renal and mineral metabolism cannot be excluded, and our findings should not be interpreted as establishing a renal-function-independent or mechanistic role of FGF23. Furthermore, given the limited number of participants with eGFR < 30 mL/min/1.73 m^2^, findings in this subgroup should be considered exploratory and interpreted with caution. Second, the observed improvements in discrimination and reclassification indices provide statistical evidence of incremental prognostic performance within this derivation cohort. However, these findings do not establish clinical utility, which requires independent external validation and assessment of their impact on clinical decision-making. Third, the available data do not allow us to clarify the underlying biological mechanisms that may explain the observed associations. Fourth, the median concentration of 48.4 pg/mL used in descriptive summaries was cohort-derived and does not represent an established biological or clinical decision threshold. Fifth, although our primary findings rely on continuous modeling across the full biomarker spectrum, defining and externally validating clinically actionable thresholds will be required for practical bedside implementation. Sixth, this study included an older Spanish cohort, which may limit the generalizability of our findings to other HF populations. Lastly, future studies, including more detailed echocardiographic evaluation and external validation in independent cohorts, are needed to validate these findings and better understand their potential clinical implications.

## 5. Conclusions

Elevated circulating FGF23 levels are independently associated with an increased risk of long-term adverse outcomes in patients with WHF. These findings support the prognostic relevance of FGF23 in patients with WHF. External validation in independent populations is warranted, and whether FGF23 has a causal pathophysiological role or represents a potential therapeutic target requires further investigation in experimental and interventional studies.

## Figures and Tables

**Table 1:** Baseline and biomarker characteristics of the whole sample and across FGF23 median.

	FGF23 ≤ 48.4 pg/mL (n = 317)	FGF23 > 48.4 pg/mL (n = 318)	Total Population (n = 635)	*p*-Value
Age, years	82.2 ± 9.9	82.6 ± 10.1	82.4 ± 10	0.639
Women, n (%)	178 (56.2)	161 (50.6)	339 (53.4)	0.163
Prior history of HF, n (%)	188 (59.3)	213 (67)	401 (63.1)	0.045
Hypertension, n (%)	263 (83)	271 (85.2)	534 (84.1)	0.437
Diabetes Mellitus, n (%)	115 (36.3)	133 (41.8)	248 (39.1)	0.152
CKD, n (%)	63 (19.9)	125 (39.3)	188 (29.6)	<0.001
Stroke, n (%)	37 (11.7)	45 (14.2)	82 (12.9)	0.352
Dyslipidemia, n (%)	128 (40.4)	148 (46.5)	276 (43.5)	0.117
Third heart sound, n (%)	5 (1.6)	8 (2.5)	13 (2)	0.404
Ischemic heart disease, n (%)	65 (20.5)	96 (30.2)	161 (25.4)	0.005
Crackles, n (%)	206 (65)	216 (67.9)	422 (66.5)	0.433
Valvular heart disease, n (%)	83 (26.2)	106 (33.3)	189 (29.8)	0.049
COPD, n (%)	79 (24.9)	59 (18.6)	138 (21.7)	0.052
LVEF, %	54.6 ± 13.3	49.9 ± 14.0	52.1 ± 13.9	0.001
Barthel index	81.8 ± 24.2	79.5 ± 24.8	80.6 ± 24.5	0.228
MEESSI Score	−2.9 ± 1.1	−2.7 ± 1.1	−2.8 ± 1.1	0.043
Heart rate, beats/min	89.3 ± 23	86.6 ± 24.2	87.9 ± 23.7	0.163
SBP, mmHg	139.9 ± 27.2	136.5 ± 26.3	138.2 ± 26.8	0.108
DBP, mmHg	75.7 ± 17.7	75.9 ± 17.3	75.8 ± 17.5	0.881
Peripheral edema, n (%)	218 (68.8)	217 (68.2)	435 (68.5)	0.886
Pleural effusion, n (%)	70 (22.1)	121 (38.1)	191 (30.1)	<0.001
Prior history of atrial fibrillation, n (%)	156 (49.2)	188 (59.1)	344 (54.2)	0.012
PAD, n (%)	22 (6.9)	37 (11.6)	59 (9.3)	0.042
Orthopnea, n (%)	180 (56.8)	161 (50.6)	341 (53.7)	0.120
Paroxysmal nocturnal dyspnea, n (%)	91 (28.7)	84 (26.4)	175 (27.6)	0.518
Jugular venous distention, n (%)	57 (18.0)	83 (26.1)	140 (22.0)	0.014
Hospital admission, n (%)	224 (70.7)	235 (73.9)	459 (72.3)	0.362
**Electrocardiogram**				
Atrial fibrillation, n (%)	150 (47.3)	167 (52.5)	317 (49.9)	0.190
**Circulating biomarkers**				
Hemoglobin, g/dL ^1^	12 (11–13.8)	11.7 (10.2–13.2)	12 (10.6–13.4)	0.005
NT-proBNP, pg/ml ^1^	3700 (1841–7319)	4838 (2714–9139)	4206.5 (2280–8420)	0.003
Serum Creatinine, mg/dL ^1^	1.1 (0.9–1.4)	1.3 (1–1.7)	1.2 (0.9–1.6)	<0.001
eGFR, mL/min/1.73 m^2^	65.2 ± 24.7	55.1 ± 25.8	60.1 ± 25.7	<0.001
Sodium, mmol/L	139.1 ± 4.8	139.5 ± 4.9	139.3 ± 4.8	0.252
CA125, U/mL ^1^	37 (16–81)	53 (21–103)	44 (19–94)	0.055
FGF23, pg/mL ^1^	27.3 (17–36)	88.1 (66–135)	48.4 (27–88)	<0.001
**HF treatment**				
Thiazides, n (%)	54 (17.0)	42 (13.2)	96 (15.1)	0.178
Beta-blockers, n (%)	117 (36.9)	160 (50.3)	277 (43.6)	0.001
ACEI, n (%)	100 (31.5)	91 (28.6)	191 (30.1)	0.421
MRA, n (%)	43 (13.6)	52 (16.4)	95 (15.0)	0.325
Loop diuretics, n (%)	197 (62.1)	222 (69.8)	419 (66.0)	0.041
ARB, n (%)	78 (24.6)	84 (26.4)	162 (25.5)	0.601
**Event rates per 100 person-years according to FGF23 categories**				
All-cause mortality, IR (95% CI)	31 (25–39)	49 (41–58)	39 (35–45)	0.003
Death/WHF ED reconsultation, IR (95% CI)	65 (59–76)	93 (81–107)	78 (70–87)	0.003
Death/HF hospitalization, IR (95% CI)	56 (47–66)	77 (66–90)	66 (59–74)	0.043

^1^ Value expressed as the median (percentile 25–percentile 75). Bold text indicates variable categories. ACEI = angiotensin-converting enzyme inhibitor; ARB = angiotensin II receptor blocker; CI = confidence interval; CKD = chronic kidney disease; CA125 = antigen carbohydrate 125; COPD = chronic obstructive pulmonary disease; DBP = diastolic blood pressure; eGFR = glomerular filtration rate estimated; ED = emergency department; FGF23 = fibroblast growth factor 23; HF = heart failure; IR = incidence rate; LVEF = left ventricular ejection fraction; NT-proBNP = N-terminal pro B-type natriuretic peptide; MRA = mineralocorticoid receptor antagonists; PAD = peripheral artery disease; SBP = systolic blood pressure; WHF = worsening heart failure.

**Table 2:** Comprehensive association of circulating FGF23 with clinical outcomes.

Endpoint and FGF23 Analytical Specification	Patients (n)	Events (%)	Unadjusted HR (95% CI)	*p*-Value	Fully Adjusted HR (95% CI) *	*p*-Value
**All-cause mortality (216 events/635 patients)**						
≤48.4 pg/mL (Median Low, Reference)	317	91 (28.7)	1.00 (Reference)		1.00 (Reference)	
>48.4 pg/mL (Median High)	318	125 (39.3)	1.50 (1.14–1.96)	0.004	1.25 (0.93–1.67)	0.143
Q1: ≤27.3 pg/mL (Reference)	159	48 (30.2)	1.00 (Reference)		1.00 (Reference)	
Q2: 27.4 to 48.4 pg/mL	159	43 (27.0)	0.86 (0.57–1.30)	0.466	0.81 (0.53–1.23)	0.321
Q3: 48.5 to 88.2 pg/mL	159	46 (28.9)	0.95 (0.63–1.42)	0.806	0.84 (0.55–1.28)	0.412
Q4: >88.2 pg/mL	158	79 (50.0)	1.91 (1.33–2.73)	<0.001	1.46 (0.98–2.17)	0.061
*p* for linear trend (per quartile increment)	635	216	1.26 (1.12–1.43)	<0.001	1.15 (1.00–1.31)	0.047
Continuous: Per doubling (log2 scale)	635	216 (34.0)	1.28 (1.16–1.41)	<0.001	1.17 (1.05–1.31)	0.004
Continuous: Per 1-SD increase in log2 (FGF23)	635	216 (34.0)	1.40 (1.23–1.59)	<0.001	1.24 (1.07–1.43)	0.004
**Death/WHF ED reconsultation (351 events/635 patients)**						
≤48.4 pg/mL (Median Low, Reference)	317	156 (49.2)	1.00 (Reference)		1.00 (Reference)	
>48.4 pg/mL (Median High)	318	195 (61.3)	1.37 (1.11–1.69)	0.004	1.29 (1.03–1.62)	0.029
Q1: ≤27.3 pg/mL (Reference)	159	76 (47.8)	1.00 (Reference)		1.00 (Reference)	
Q2: 27.4 to 48.4 pg/mL	159	81 (50.9)	1.09 (0.79–1.49)	0.605	1.08 (0.78–1.49)	0.654
Q3: 48.5 to 88.2 pg/mL	159	85 (53.5)	1.15 (0.84–1.56)	0.384	1.12 (0.80–1.55)	0.510
Q4: >88.2 pg/mL	158	109 (69.0)	1.74 (1.29–2.33)	<0.001	1.59 (1.16–2.20)	0.005
*p* for linear trend (per quartile increment)	635	351	1.20 (1.09–1.32)	<0.001	1.16 (1.05–1.29)	0.005
Continuous: Per doubling (log2 scale)	635	351 (55.3)	1.19 (1.10–1.29)	<0.001	1.14 (1.05–1.24)	0.002
Continuous: Per 1-SD increase in log2(FGF23)	635	351 (55.3)	1.26 (1.14–1.40)	<0.001	1.20 (1.07–1.34)	0.002
**Death/HF hospitalization (295 events/635 patients)**						
≤48.4 pg/mL (Median Low, Reference)	317	134 (42.3)	1.00 (Reference)		1.00 (Reference)	
>48.4 pg/mL (Median High)	318	161 (50.6)	1.27 (1.01–1.59)	0.044	1.15 (0.90–1.48)	0.271
Q1: ≤27.3 pg/mL (Reference)	159	63 (39.6)	1.00 (Reference)		1.00 (Reference)	
Q2: 27.4 to 48.4 pg/mL	159	71 (44.7)	1.14 (0.81–1.60)	0.443	1.13 (0.80–1.60)	0.494
Q3: 48.5 to 88.2 pg/mL	159	65 (40.9)	1.04 (0.73–1.46)	0.844	1.03 (0.71–1.49)	0.876
Q4: >88.2 pg/mL	158	96 (60.8)	1.73 (1.26–2.38)	0.001	1.46 (1.03–2.07)	0.033
*p* for linear trend (per quartile increment)	635	295	1.18 (1.06–1.31)	0.002	1.12 (1.00–1.25)	0.054
Continuous: Per doubling (log2 scale)	635	295 (46.5)	1.19 (1.10–1.30)	<0.001	1.12 (1.02–1.22)	0.013
Continuous: Per 1-SD increase in log2(FGF23)	635	295 (46.5)	1.27 (1.14–1.42)	<0.001	1.17 (1.03–1.31)	0.013

* Estimates were adjusted for age, sex, prior history of HF, history of ischemic heart disease, atrial fibrillation at admission, systolic blood pressure, heart rate, Barthel index score, orthopnea, paroxysmal nocturnal dyspnea, third heart sound, jugular venous distension, pleural effusion, peripheral edema, hemoglobin, NT-proBNP and creatinine levels. Bold text indicates outcome categories. CI = confidence interval; ED = emergency department; FGF23 = fibroblast growth factor 23; HF = heart failure; HR = hazard ratio; Q = quartile; SD = standard deviation; WHF = worsening heart failure.

**Table 3:** Incremental predictive value, discrimination, and reclassification performance of circulating FGF23 beyond the multivariable clinical model across follow-up.

Clinical Endpoint	Events/Total (%)	Base Model Harrell’s C	Model + FGF23 Harrell’s C	LR Test *p*-Value	Continuous NRI (>0) [95% CI]	NRI *p*-Value	IDI [95% CI]	IDI *p*-Value
All-cause mortality	216/635 (34.0)	0.701	0.710	0.0015	0.1810 [0.0188, 0.3433]	0.0288	0.0210 [0.0081, 0.0339]	0.0014
Death/WHF ED reconsultation	351/635 (55.3)	0.637	0.639	0.0058	0.1699 [0.0156, 0.3242]	0.0309	0.0145 [0.0048, 0.0241]	0.0034
Death/HF hospitalization	295/635 (46.5)	0.650	0.652	0.0407	0.1474 [−0.0068, 0.3015]	0.0610	0.0127 [0.0033, 0.0221]	0.0079

Harrell’s C is the concordance index for time-to-event survival models across the total follow-up. LR test represents the likelihood-ratio test comparing the nested multivariable models with vs. without FGF23. Continuous NRI (>0) is the category-free Net Reclassification Improvement (sum of event and non-event net reclassification). IDI represents the Integrated Discrimination Improvement. CI = confidence interval; ED = emergency department; FGF23 = fibroblast growth factor 23; HF = heart failure; IDI = integrated discrimination improvement; LR = likelihood-ratio; NRI = net reclassification improvement; WHF = worsening heart failure.

**Table 4:** Multivariable cox proportional hazards models for circulating FGF23 across renal function, sex, and age subgroups.

Endpoint and Subgroup Stratum	Patients (n)	Events, n (%)	Adjusted HR (95% CI) *	*p*-Value	*p*-Value for Interaction
**All-cause mortality**					
**Baseline Kidney Function (eGFR CKD-EPI, mL/min/1.73 m^2^)**					
≥60 mL/min/1.73 m^2^	286	69 (24.1%)	1.11 (0.86–1.43)	0.416	0.314
30.0–59.9 mL/min/1.73 m^2^	273	104 (38.1%)	1.10 (0.94–1.30)	0.226	
<30.0 mL/min/1.73 m^2^	76	43 (56.6%)	1.28 (0.98–1.67)	0.065	
**Sex**					
Women	339	115 (33.9%)	1.17 (1.01–1.37)	0.042	0.424
Men	296	101 (34.1%)	1.06 (0.90–1.24)	0.499	
**Age Category (years)**					
<80 years	194	53 (27.3%)	1.23 (1.00–1.51)	0.054	0.274
≥80 years	441	163 (37.0%)	1.11 (0.97–1.27)	0.146	
**Death/WHF ED reconsultation**					
**Baseline Kidney Function (eGFR CKD-EPI, mL/min/1.73 m^2^)**					
≥60 mL/min/1.73 m^2^	286	130 (45.5%)	1.13 (0.95–1.34)	0.173	0.142
30.0–59.9 mL/min/1.73 m^2^	273	169 (61.9%)	1.06 (0.93–1.20)	0.385	
<30.0 mL/min/1.73 m^2^	76	52 (68.4%)	1.35 (1.06–1.72)	0.014	
**Sex**					
Women	339	195 (57.5%)	1.20 (1.07–1.35)	0.003	0.745
Men	296	156 (52.7%)	1.06 (0.93–1.20)	0.389	
**Age Category (years)**					
<80 years	194	90 (46.4%)	1.14 (0.97–1.34)	0.119	0.801
≥80 years	441	261 (59.2%)	1.12 (1.00–1.24)	0.045	
**Death/HF hospitalization**					
**Baseline Kidney Function (eGFR CKD-EPI, mL/min/1.73 m^2^)**					
≥60 mL/min/1.73 m^2^	286	104 (36.4%)	1.06 (0.88–1.28)	0.552	0.463
30.0–59.9 mL/min/1.73 m^2^	273	142 (52.0%)	1.07 (0.94–1.23)	0.303	
<30.0 mL/min/1.73 m^2^	76	49 (64.5%)	1.27 (1.00–1.62)	0.049	
**Sex**					
Women	339	157 (46.3%)	1.19 (1.04–1.36)	0.010	0.613
Men	296	138 (46.6%)	1.02 (0.90–1.17)	0.724	
**Age Category (years)**					
<80 years	194	72 (37.1%)	1.05 (0.88–1.26)	0.559	0.984
≥80 years	441	223 (50.6%)	1.11 (0.99–1.25)	0.065	

* Adjusted Hazard Ratios are reported per doubling (log2 increase) of circulating FGF23. Each stratum-specific model was adjusted for the remaining 25 pre-specified baseline covariates (excluding the stratifying factor). Bold text indicates outcome and subgroup categories. CI = confidence interval; CKD-EPI = chronic kidney disease epidemiology collaboration; ED = emergency department; eGFR = estimated glomerular filtration rate; FGF23 = fibroblast growth factor 23; FP = flexible parametric spline model; HF = heart failure; HR = hazard ratio; WHF = worsening heart failure.

## Data Availability

The data used in this study are derived from patients and contain sensitive clinical information. They may be made available upon reasonable request to the corresponding author, subject to the applicable ethical and privacy requirements.

## Abbreviations

The following abbreviations are used in this manuscript:

WHF	Worsening heart failure
FGF23	Fibroblast growth factor 23
ED	Emergency department
CV	Cardiovascular
CKD	Chronic kidney disease
HF	Heart failure
EAHFE	Epidemiology of Acute Heart Failure in Spanish Emergency Departments
IQR	Interquartile range
SD	Standard deviation
IR	Incidence rate
eGFR	Estimated glomerular filtration rate
VIF	Variance inflation factors
MFP	Multivariable fractional polynomials
AIC	Akaike information criterion
IDI	Integrated discrimination improvement
NRI	Net reclassification improvement
HR	Hazard ratio
MICE	Multiple Imputation by Chained Equations
CI	Confidence interval
LVEF	Left ventricular ejection fraction

## Appendix A

**Table A1:** Baseline characteristics of the full cohort according to FGF23 measurement availability.

Variable	Full Cohort (N = 764)	Valid Biomarkers (n = 635, 83.1%)	Missing Biomarkers (n = 129, 16.9%)	Missing Data in Cohort, n (%)	*p*-Value *
Age, years (mean ± SD)	82.2 ± 9.8	82.4 ± 10.0	81.5 ± 9.1	0 (0.0)	0.358
Female sex, n (%)	415 (54.3)	339 (53.4)	76 (58.9)	0 (0.0)	0.250
SBP, mmHg (mean ± SD)	138.4 ± 27.7	138.2 ± 26.8	139.0 ± 31.7	0 (0.0)	0.771
Heart rate, bpm (mean ± SD)	88.1 ± 24.0	87.9 ± 23.7	88.8 ± 25.6	0 (0.0)	0.701
Atrial fibrillation, n (%)	390 (51.0)	317 (49.9)	69 (53.5)	0 (0.0)	0.546
Barthel Index (mean ± SD)	78.6 ± 26.3	78.4 ± 26.5	79.4 ± 25.2	0 (0.0)	0.692
Hypertension, n (%)	639 (83.6)	534 (84.1)	108 (83.7)	0 (0.0)	0.978
Diabetes mellitus, n (%)	332 (43.5)	248 (39.1)	55 (42.6)	0 (0.0)	0.835
Prior history of HF, n (%)	482 (63.1)	401 (63.1)	81 (62.8)	0 (0.0)	0.939
eGFR, mL/min/1.73 m^2^ (mean ± SD)	60.4 ± 25.9	60.1 ± 25.7	61.8 ± 26.6	0 (0.0)	0.500
Hemoglobin, g/dL (mean ± SD)	12.0 ± 2.2	12.0 ± 2.2	11.8 ± 2.2	1 (0.1)	0.423
Serum sodium, mEq/L (mean ± SD)	138.7 ± 5.1	139.3 ± 4.9	138.8 ± 5.4	0 (0.0)	0.812
FGF23, pg/mL (median [IQR])	—	48.4 (27.3–88.2)	Missing	129 (16.9)	—
Outcomes:					
All-cause mortality, n (%)	265 (34.7)	216 (34.0)	49 (38.0)	0 (0.0)	0.388
Death/WHF ED reconsultation, n (%)	417 (55.3)	351 (55.3)	66 (55.5)	10 (1.3)	0.970
Death/HF hospitalization, n (%)	359 (47.0)	295 (46.5)	64 (49.6)	0 (0.0)	0.513

* *p*-values calculated using Student’s *t*-test for continuous normally distributed variables, Mann–Whitney U test for skewed variables, and Pearson’s chi-square test for categorical variables comparing included vs. excluded patients. eGFR = glomerular filtration rate estimated; ED = emergency department; FGF23 = fibroblast growth factor 23; HF = heart failure; IQR = interquartile range; SBP = systolic blood pressure; SD = standard deviation; WHF = worsening heart failure.

**Table A2:** Baseline characteristics across FGF23 quartiles.

	FGF23 Quartile 1 (≤27.3 pg/mL) (n = 159)	FGF23 Quartile 2 (27.4–48.4 pg/mL) (n = 159)	FGF23 Quartile 3 (48.5–88.2 pg/mL) (n = 159)	FGF23 Quartile 4 (>88.2 pg/mL) (n = 158)	*p*-Value
Age, years	82.1 ± 9.4	82.3 ± 10.3	81.9 ± 10.4	83.3 ± 9.7	0.621
Women, n (%)	90 (57)	88 (55.3)	82 (51.6)	79 (49.7)	0.543
Prior history of HF, n (%)	92 (58.2)	96 (60.4)	98 (61.6)	115 (72.3)	0.044
Hypertension, n (%)	135 (85.4)	128 (80.5)	135 (84.9)	136 (85.5)	0.557
Diabetes Mellitus, n (%)	60 (38.0)	55 (34.6)	69 (43.4)	64 (40.3)	0.430
CKD, n (%)	30 (19)	33 (20.8)	47 (29.6)	78 (49.1)	<0.001
Stroke, n (%)	13 (8.2)	24 (15.1)	27 (17)	18 (11.3)	0.091
Dyslipidemia, n (%)	61 (38.6)	67 (42.1)	80 (50.3)	68 (42.8)	0.195
Ischemic heart disease, n (%)	31 (19.6)	34 (21.4)	47 (29.6)	49 (30.8)	0.045
Crackles, n (%)	102 (64.6)	104 (65.4)	102 (64.2)	114 (71.7)	0.445
Third heart sound, n (%)	4 (2.5)	1 (0.6)	2 (1.3)	6 (3.8)	0.200
Valvular heart disease, n (%)	49 (31)	34 (21.4)	48 (30.2)	58 (36.5)	0.031
COPD, n (%)	37 (23.4)	42 (26.4)	32 (20.1)	27 (17)	0.198
LVEF, %	57.2 ± 12.4	52.1 ± 13.8	49.9 ± 14.0	50.0 ± 14.1	<0.001
Barthel index	83.8 ± 21.9	79.8 ± 26.2	78.9 ± 26.2	80.1 ± 23.2	0.283
MEESSI Score	−2.8 ± 1.2	−2.9 ± 1.1	−2.8 ± 1.1	−2.5 ± 1.1	0.046
Heart rate, beats/min	89.1 ± 22.6	89.4 ± 23.6	87.1 ± 22.6	86.2 ± 25.8	0.553
SBP, mmHg	139.3 ± 27.4	140.6 ± 27.2	138.1 ± 25.9	134.9 ± 26.8	0.276
DBP, mmHg	74.5 ± 17.8	76.9 ± 17.6	76.7 ± 16.7	75.1 ± 17.9	0.546
Peripheral edema, n (%)	109 (69)	109 (68.6)	99 (62.3)	118 (74.2)	0.152
Pleural effusion, n (%)	26 (16.5)	44 (27.7)	57 (35.8)	64 (40.3)	<0.001
Prior history of atrial fibrillation, n (%)	83 (52.5)	73 (45.9)	93 (58.5)	95 (59.7)	0.052
PAD, n (%)	10 (6.3)	12 (7.5)	23 (14.5)	14 (8.8)	0.063
Orthopnea, n (%)	96 (60.8)	84 (52.8)	82 (51.6)	79 (49.7)	0.209
Paroxysmal nocturnal dyspnea, n (%)	45 (28.5)	46 (28.9)	47 (29.6)	37 (23.3)	0.572
Jugular venous distention, n (%)	27 (17.1)	30 (18.9)	43 (27.0)	40 (25.2)	0.094
Hospital admission, n (%)	113 (71.5)	111 (69.8)	120 (75.5)	115 (72.3)	0.720
**Electrocardiogram**					
Atrial fibrillation, n (%)	75 (47.5)	75 (47.2)	84 (52.8)	83 (52.2)	0.630
**Circulating biomarkers**					
Hemoglobin, g/dL ^1^	11.9 (10.9–13.6)	12.4 (11.1–14.1)	12.4 (10.7–13.5)	11.1 (10–12.6)	<0.001
NT-proBNP, pg/ml ^1^	4041.5 (2003.5–7638)	3641 (1718–7193)	4042 (2392–8026)	5803 (2862–13,162)	0.001
Serum Creatinine, mg/dL ^1^	1.1 (0.8–1.4)	1.1 (0.9–1.3)	1.1 (0.9–1.5)	1.4 (1.1–1.9)	<0.001
eGFR, mL/min/1.73 m^2^	64.8 ± 26.3	65.5 ± 23.2	61.8 ± 26.3	48.5 ± 23.4	<0.001
Sodium, mmol/L	139.2 ± 5.0	139.0 ± 4.5	139.4 ± 3.8	139.6 ± 5.8	0.666
CA125, U/mL ^1^	36 (17–79.5)	38 (15.5–83)	49 (20–97)	59 (22–110)	0.042
FGF23, pg/mL ^1^	16.9 (11.8–22.6)	35.8 (31.5–42.6)	66.3 (56.6–74.8)	134.6 (102.7–188.0)	<0.001
**HF treatment**					
Thiazides, n (%)	26 (16.5)	28 (17.6)	21 (13.2)	21 (13.2)	0.595
Beta-blockers, n (%)	53 (33.5)	64 (40.3)	75 (47.2)	85 (53.5)	0.002
ACEI, n (%)	53 (33.5)	47 (29.6)	42 (26.4)	49 (30.8)	0.577
MRA, n (%)	17 (10.8)	26 (16.4)	24 (15.1)	28 (17.6)	0.346
Loop diuretics, n (%)	100 (63.3)	97 (61.0)	103 (64.8)	119 (74.8)	0.048
ARB, n (%)	40 (25.3)	38 (23.9)	48 (30.2)	36 (22.6)	0.433

^1^ Value expressed as the median (percentile 25–percentile 75). Bold text indicates variable categories. ACEI = angiotensin-converting enzyme inhibitor; ARB = angiotensin II receptor blocker; CA125 = antigen carbohydrate 125; CKD = chronic kidney disease; COPD = chronic obstructive pulmonary disease; DBP = diastolic blood pressure; eGFR = glomerular filtration rate estimated; FGF23 = fibroblast growth factor 23; HF = heart failure; LVEF = left ventricular ejection fraction; NT-proBNP = N-terminal pro B-type natriuretic peptide; ED = emergency department, MRA = mineralocorticoid receptor antagonists; PAD = peripheral artery disease; SBP = systolic blood pressure.

**Table A3:** Event rates per 100 person-years according to FGF23 quartiles.

	IR (95% CI)	*p*-Value
All-cause mortality		<0.001
Quartile 1	33 (25–44)	
Quartile 2	29 (21–40)	
Quartile 3	33 (25–44)	
Quartile 4	68 (55–85)	
Death/WHF ED reconsultation		<0.001
Quartile 1	61 (49–77)	
Quartile 2	69 (56–86)	
Quartile 3	74 (60–92)	
Quartile 4	117 (98–142)	
Death/HF hospitalization		0.001
Quartile 1	51 (40–65)	
Quartile 2	62 (49–78)	
Quartile 3	56 (44–72)	
Quartile 4	103 (85–127)	

Incidence rates with 95% confidence intervals and *p*-values are presented of all-cause mortality, death/WHF ED reconsultation and death/HF hospitalization, stratified by FGF23 levels across quartiles. CI = confidence interval; ED = emergency department; IR = incidence rate; HF = heart failure; WHF = worsening heart failure.

**Table A4:** FGF23 and adverse outcomes. Unadjusted and adjusted estimates.

Model Specification	All-Cause Mortality Adj. HR (95% CI), *p*-Value	Death/WHF ED Reconsultation Adj. HR (95% CI), *p*-Value	Death/HF Hosp Adj. HR (95% CI), *p*-Value
Unadjusted Model (per doubling)	1.28 (1.16–1.41), *p* < 0.001	1.19 (1.10–1.29), *p* < 0.001	1.19 (1.10–1.30), *p* < 0.001
Demographics + Comorbidities	1.25 (1.13–1.39), *p* < 0.001	1.17 (1.08–1.28), *p* < 0.001	1.16 (1.06–1.27), *p* = 0.001
Final multivarite model	1.17 (1.05–1.31), *p* = 0.004	1.14 (1.05–1.24), *p* = 0.002	1.12 (1.02–1.22), *p* = 0.013
MFP-Selected Model (*p* < 0.20 /AIC)	1.18 (1.06–1.32), *p* = 0.003	1.15 (1.06–1.25), *p* = 0.001	1.13 (1.03–1.23), *p* = 0.010

Hazard ratios (HR) and 95% confidence intervals (CI) represent the relative risk associated with a doubling of circulating FGF23 concentration (${log}_{2}$ scale). Unadjusted Model: Evaluates log_2_ (FG23) as a single continuous predictor. Demographics + Comorbidities Model (n = 11 covariates): Adjusted for age, sex, baseline Barthel Index score, coronary artery disease, hypertension, diabetes mellitus, dyslipidemia, previous stroke, chronic obstructive pulmonary disease (COPD), prior history of heart failure, and valvular heart disease. Final Multivariable Model included age, sex, prior history of HF, history of ischemic heart disease, atrial fibrillation at admission, systolic blood pressure, heart rate, Barthel index score, orthopnea, paroxysmal nocturnal dyspnea, third heart sound, jugular venous distension, pleural effusion, peripheral edema, hemoglobin, NT-proBNP and creatinine levels MFP-Selected Model (Multivariable Fractional Polynomials): Parsimonious models identified using backward elimination with a retention threshold of α = 0.156–0.20 (asymptotically equivalent to optimizing the Akaike Information Criterion, AIC, to avoid post-selection coefficient shrinkage and omitted-variable bias). The specific covariates retained in the final MFP equation for each endpoint were: All-Cause Mortality (*n* = 10 covariates)*:* Age, sex, systolic blood pressure (FP power—2), heart rate (FP powers 2,3), Barthel Index, peripheral edema, third heart sound, eGFR, CA125, and FGF23 (FP powers—0.5,0). Death or WHF ED Reconsultation (*n* = 11 covariates): Age (FP powers—2,0), systolic blood pressure, atrial fibrillation, Barthel Index, coronary artery disease, hypertension, dyslipidemia, prior heart failure, serum sodium, CA125, and FGF23 (log transformation, power 0). Death or HF Hospitalization (n = 13 covariates): Age (FP powers—2,2), sex, systolic blood pressure, Barthel Index, coronary artery disease, hypertension, dyslipidemia, prior heart failure, orthopnea, hemoglobin, serum sodium, CA125, and FGF23 (log transformation, power 0). CI = confidence interval; ED = emergency department; HR = hazard ratio; HF = heart failure; MFP = multivariable fractional polynomials; WHF = worsening heart failure.

## References

[1] Arrigo M., Jessup M., Mullens W., Reza N., Shah A.M., Sliwa K., Mebazaa A. (2020). Acute heart failure. Nat. Rev. Dis. Primers.

[2] Greene S.J., Bauersachs J., Brugts J.J., Ezekowitz J.A., Lam C.S.P., Lund L.H., Ponikowski P., Voors A.A., Zannad F., Zieroth S. (2023). Worsening Heart Failure: Nomenclature, Epidemiology, and Future Directions: JACC Review Topic of the Week. JACC.

[3] Kittipibul V., Fudim M., Sobotka P.A. (2023). Congestion and inflammation in heart failure: Beyond the chicken or the egg. J. Card. Fail..

[4] Amrute J.M., Luo X., Penna V., Yang S., Yamawaki T., Hayat S., Bredemeyer A., Jung I.H., Kadyrov F.F., Heo G.S. (2024). Targeting immune–fibroblast cell communication in heart failure. Nature.

[5] Joury A., Ventura H., Krim S.R. (2022). Biomarkers in heart failure: Relevance in the clinical practice. Int. J. Cardiol..

[6] Brann A., Selko S., Krauspe E., Shah K. (2024). Biomarkers of Hemodynamic Congestion in Heart Failure. Curr. Heart Fail. Rep..

[7] Martínez-Heredia L., Canelo-Moreno J.M., García-Fontana B., Muñoz-Torres M. (2024). Non-Classical Effects of FGF23: Molecular and Clinical Features. Int. J. Mol. Sci..

[8] Ter Maaten J.M., Voors A.A., Damman K., van der Meer P., Anker S.D., Cleland J.G., Dickstein K., Filippatos G., van der Harst P., Hillege H.L. (2018). Fibroblast growth factor 23 is related to profiles indicating volume overload, poor therapy optimization and prognosis in patients with new-onset and worsening heart failure. Int. J. Cardiol..

[9] Martí-Martínez A., Núñez J., López-Escribano H., Revuelta-López E., Mollar A., Peiró M., Sanchis J., Bayés-Genís A., Carratala A., Miró Ò. (2025). The Role of Antigen Carbohydrate 125 in Modulating Soluble ST2: Prognostic-Related Effects in Acute Heart Failure. Biomolecules.

[10] Llorens P., Escoda R., Miró Ò., Herrero-Puente P., Martín-Sánchez F.J., Jacob J., Garrido J.M., Pérez-Durá M.J., Gil C., Fuentes M. (2015). Characteristics and clinical course of patients with acute heart failure and the therapeutic measures applied in Spanish emergency departments: Based on the EAHFE registry (Epidemiology of Acute Heart Failure in Emergency Departments). Emergencias.

[11] Goetz R., Nakada Y., Hu M.C., Kurosu H., Wang L., Nakatani T., Shi M., Eliseenkova A.V., Razzaque M.S., Moe O.W. (2010). Isolated C-terminal tail of FGF23 alleviates hypophosphatemia by inhibiting FGF23-FGFR-Klotho complex formation. Proc. Natl. Acad. Sci. USA.

[12] Pencina M.J., D’Agostino R.B., D’Agostino R.B., Vasan R.S. (2008). Evaluating the added predictive ability of a new marker: From area under the ROC curve to reclassification and beyond. Stat. Med..

[13] White I.R., Royston P., Wood A.M. (2011). Multiple imputation using chained equations: Issues and guidance for practice. Stat. Med..

[14] Vázquez-Sánchez S., Poveda J., Navarro-García J.A., González-Lafuente L., Rodríguez-Sánchez E., Ruilope L.M., Ruiz-Hurtado G. (2021). An Overview of FGF-23 as a Novel Candidate Biomarker of Cardiovascular Risk. Front. Physiol..

[15] Vergaro G., Del Franco A., Aimo A., Gentile F., Castiglione V., Saponaro F., Masotti S., Prontera C., Fusari N., Emdin M. (2023). Intact fibroblast growth factor 23 in heart failure with reduced and mildly reduced ejection fraction. BMC Cardiovasc. Disord..

[16] Wang T.J. (2011). Assessing the role of circulating, genetic, and imaging biomarkers in cardiovascular risk prediction. Circulation.

[17] Navarro-García J.A., González-Lafuente L., Fernández-Velasco M., Ruilope L.M., Ruiz-Hurtado G. (2021). Fibroblast Growth Factor-23-Klotho Axis in Cardiorenal Syndrome: Mediators and Potential Therapeutic Targets. Front. Physiol..

[18] Faul C., Amaral A.P., Oskouei B., Hu M.C., Sloan A., Isakova T., Gutiérrez O.M., Aguillon-Prada R., Lincoln J., Hare J.M. (2011). FGF23 induces left ventricular hypertrophy. J. Clin. Investig..

[19] Andrukhova O., Slavic S., Smorodchenko A., Zeitz U., Shalhoub V., Lanske B., Pohl E.E., Erben R.G. (2014). FGF23 regulates renal sodium handling and blood pressure. EMBO Mol. Med..

[20] Poelzl G., Trenkler C., Kliebhan J., Wuertinger P., Seger C., Kaser S., Mayer G., Pirklbauer M., Ulmer H., Griesmacher A. (2014). FGF23 is associated with disease severity and prognosis in chronic heart failure. Eur. J. Clin. Investig..

[21] Koller L., Kleber M.E., Brandenburg V.M., Goliasch G., Richter B., Sulzgruber P., Scharnagl H., Silbernagel G., Grammer T.B., Delgado G. (2015). Fibroblast Growth Factor 23 Is an Independent and Specific Predictor of Mortality in Patients With Heart Failure and Reduced Ejection Fraction. Circ. Heart Fail..

[22] Kanagala P., Arnold J.R., Khan J.N., Singh A., Gulsin G.S., Eltayeb M., Gupta P., Squire I.B., McCann G.P., Ng L.L. (2020). Fibroblast-growth-factor-23 in heart failure with preserved ejection fraction: Relation to exercise capacity and outcomes. ESC Heart Fail..

[23] Roy C., Lejeune S., Slimani A., de Meester C., Ahn As S.A., Rousseau M.F., Mihaela A., Ginion A., Ferracin B., Pasquet A. (2020). Fibroblast growth factor 23: A biomarker of fibrosis and prognosis in heart failure with preserved ejection fraction. ESC Heart Fail..

[24] Vergaro G., Aimo A., Taurino E., Del Franco A., Fabiani I., Prontera C., Masotti S., Musetti V., Emdin M., Passino C. (2021). Discharge FGF23 level predicts one year outcome in patients admitted with acute heart failure. Int. J. Cardiol..

[25] Fuernau G., Pöss J., Denks D., Desch S., Heine G.H., Eitel I., Seiler S., de Waha S., Ewen S., Link A. (2014). Fibroblast growth factor 23 in acute myocardial infarction complicated by cardiogenic shock: A biomarker substudy of the Intraaortic Balloon Pump in Cardiogenic Shock II (IABP-SHOCK II) trial. Crit. Care.

[26] Miró Ò., Rossello X., Platz E., Masip J., Gualandro D.M., Peacock W.F., Price S., Cullen L., DiSomma S., de Oliveira M.T. (2020). Study Group on Acute Heart Failure of the Acute Cardiovascular Care Association of the European Society of Cardiology. Risk stratification scores for patients with acute heart failure in the Emergency Department: A systematic review. Eur. Heart J. Acute Cardiovasc. Care.

[27] Lazzarini V., Mentz R.J., Fiuzat M., Metra M., O’Connor C.M. (2013). Heart failure in elderly patients: Distinctive features and unresolved issues. Eur. J. Heart Fail..

[28] Ho B.B., Bergwitz C. (2021). FGF23 signalling and physiology. J. Mol. Endocrinol..

[29] Latic N., Erben R.G. (2022). Interaction of Vitamin D with Peptide Hormones with Emphasis on Parathyroid Hormone, FGF23, and the Renin-Angiotensin-Aldosterone System. Nutrients.

[30] Nakano T., Kishimoto H., Tokumoto M. (2023). Direct and indirect effects of fibroblast growth factor 23 on the heart. Front. Endocrinol..

[31] Lee T.W., Chung C.C., Lee T.I., Lin Y.K., Kao Y.H., Chen Y.J. (2022). Fibroblast Growth Factor 23 Stimulates Cardiac Fibroblast Activity through Phospholipase C-Mediated Calcium Signaling. Int. J. Mol. Sci..

[32] Leifheit-Nestler M., Haffner D. (2018). Paracrine Effects of FGF23 on the Heart. Front. Endocrinol..

[33] Santas E., Martí-Martínez A., Villar S., de la Espriella R., Rodriguez-Borja E., Revuelta-López E., González-Miqueo A., Bayés-Genís A., Sanchis J., Núñez J. (2025). Cytokine Networks and Heart Failure Outcomes: CA125 as a Bridge Between Congestion and Inflammation. Int. J. Mol. Sci..

[34] Richter B., Faul C. (2018). FGF23 Actions on Target Tissues-With and Without Klotho. Front. Endocrinol..

